# BIOLOGICAL AND SOCIAL DETERMINANTS OF LATE-LIFE DISABILITY AND ITS IMPLICATIONS FOR AGING WORKFORCE

**DOI:** 10.1093/geroni/igad104.1006

**Published:** 2023-12-21

**Authors:** Yuan Zhang, Kate Duchowny

## Abstract

As the population continues to age, the number of people living with disability is increasing. Disability prevention is crucial for independent living and quality of life for older adults. Identifying biological, individual, and social factors that can delay the disablement process has significant policy and public health implications. This symposium comprises four papers examining critical determinants of old-age disability and the implications for the aging workforce. These papers use data from nationally representative biosocial surveys and cover a range of biological and social factors, including infection, immune aging, gender, social relationships, and work environment. First, Duchowny and Noppert will present findings on the association between advanced immune aging and physical disability in men and women. Their results suggest that immunosenescence may be a critical factor underlying gender differences in disability. Second, He will present her work on estimating the fraction of disability attributable to Cytomegalovirus (CMV) infection, highlighting the potential benefits of a vaccine for CMV in preventing disability. Third, Zhang will present results from a longitudinal study examining the pathways among various aspects of social relationships, physical limitations, and disability risk. Finally, Abrams will present educational disparities in health-induced work limitations and the importance of work characteristics in explaining such disparities. Together, the four studies presented in this symposium will enhance our understanding of determinants of the disablement process and provide valuable insights that can inform policies and interventions aimed at reducing disability and maintaining the productivity and well-being of older adults.

